# Epidemiology of smoking among Malaysian adult males: prevalence and associated factors

**DOI:** 10.1186/1471-2458-13-8

**Published:** 2013-01-07

**Authors:** Hock Kuang Lim, Sumarni Mohd Ghazali, Cheong Chee Kee, Kuay Kuang Lim, Ying Ying Chan, Huey Chien Teh, Ahmad Faudzi Mohd Yusoff, Gurpreet Kaur, Zarihah Mohd Zain, Mohamad Haniki Nik Mohamad, Sallehuddin Salleh

**Affiliations:** 1 Proposal Development Section, Institute of Public Health, Jalan Bangsar, 50590, Kuala Lumpur, Malaysia; 2 Epidemiology and Biostatistic unit, Institute for Medical Research, Jalan Pahang, Kuala Lumpur, Malaysia; 3 Disease Control Division, Ministry of Health, 62590, Putrajaya, Malaysia; 4 Pharmacy Practice Department, International Islamic University Malaysia, Jalan Sultan Ahmad Shah, Bandar Indera Mahkota, 25200, Kuantan, Pahang, Malaysia; 5 Health Division, Kuala Lumpur City Hall, Jalan Raja Laut, 50350, Kuala Lumpur, Malaysia

**Keywords:** Smoking, Malaysian adult males, Prevalence, Socio-demographic factors

## Abstract

**Background:**

Three National Health and Morbidity Surveys (NHMSs) had been conducted in Malaysia in 10-year intervals from 1986–2006. Based on the latest NHMS survey in 2006, we describe the prevalence of smoking and identify the social and demographic factors associated with smoking among adult males in Malaysia.

**Methods:**

A cross-sectional study among 15,639 Malaysian adult males aged 18 years and above was conducted using proportional to size stratified sampling method. The socio-demographic variables examined were level of education, occupation, marital status, residential area, age group and monthly household income.

**Results:**

The prevalence of smoking among adult males in Malaysia was 46.5% (95% CI: 45.5–47.4%), which was 3% lower than a decade ago. Mean age of smoking initiation was 18.3 years, and mean number of cigarettes smoked daily was 11.3. Prevalence of smoking was highest among the Malays (55.9%) and those aged 21–30 years (59.3%). Smoking was significantly associated with level of education (no education OR 2.09 95% CI (1.67–2.60), primary school OR 1.95, 95% CI (1.65–2.30), secondary school OR 1.88, 95% CI (1.63–2.11), with tertiary education as the reference group). Marital status (divorce OR 1.67, 95% CI (1.22–2.28), with married as the reference group), ethnicity (Malay, OR 2.29, 95% CI ( 1.98–2.66; Chinese OR 1.23 95% CI (1.05–1.91), Other Bumis OR 1.75, 95% CI (1.46–2.10, others OR 1.48 95% CI (1.15–1.91), with Indian as the reference group), age group (18–20 years OR 2.36, 95% CI (1.90–2.94); 20–29 years OR 3.31 , 95% CI 2.82–3.89; 31–40 years OR 2.85 , 95% CI ( 2.47–3.28); 41–50 years OR 1.93, 95% CI (1.69–2.20) ; 51–60 years OR 1.32, 95% CI (1.15–1.51), with 60 year-old and above as the reference group) and residential area (rural OR 1.12 , 95% CI ( 1.03–1.22)) urban as reference.

**Conclusion:**

The prevalence of smoking among Malaysian males remained high in spite of several population interventions over the past decade. Tobacco will likely remain a primary cause of premature mortality and morbidity in Malaysia. Continuous and more comprehensive anti-smoking policy measures are needed in order to further prevent the increasing prevalence of smoking among Malaysian men, particularly those who are younger, of Malay ethnicity, less educated, reside in rural residential area and with lower socio-economic status.

## Background

Smoking-related diseases such as cancer and cardiovascular disease are the main cause of premature death globally [1]. The current global estimate of 1.3 billion smokers is expected to increase to 1.6 billion by 2025, and the number of deaths due to smoking-related diseases is expected to reach 8.3 million by 2030, up from 4.8 million in 2006 [2]. As developing countries comprise 73% of the world’s smoker population, these countries will be more adversely affected by the health, economic and social impacts of smoking-related diseases [3-5]. In Malaysia, smoking-related diseases have been the primary cause of mortality for the past three decades [6]. A study on the burden of disease in 2003 estimated that one-fifth of disability adjusted life years (DALYs) and one-third of years of life lost (YLL) for Malaysians were due to smoking-related diseases [7]. Moreover, the country has spent as much as 2.92 billion Malaysian ringgit treating chronic obstructive pulmonary disease, ischemic heart disease and lung cancer [8]. As a result, the country seeks to cut the current smoking prevalence into half by year 2020 [9].

The tobacco industry in Malaysia is estimated to be worth more than USD 2 billion a year with three major multinational companies dominating the industry [10,11]. Besides the legal tobacco trade, illicit cigarette trade has also proliferated, accounting for approximately one fifth of the tobacco market in Malaysia [11]. To reduce the consumption of tobacco product among Malaysians, the Malaysian government has instituted many anti-smoking measures. These include introduction of tobacco control regulations in 1993, prohibition of advertisement on tobacco products and event sponsorship from tobacco companies, and control on the sales of tobacco products to minors. However, local and foreign tobacco industries circumvent such control efforts through trademark diversification. For example, advertising a tobacco brand on a non-tobacco product or selling non-tobacco products carrying tobacco brand names [12]. Hence, amendments were made to the tobacco control regulations 1993 to ban all advertisements of tobacco brands on the non-tobacco product. However, indirect advertising of cigarette brands in display cabinets may still be found at the check-out counters of sundry shops, food outlets and supermarkets [13].

Besides, other measures such as designation of smoke-free areas in 2004 [14], restructuring of tobacco taxes to increase the cost of cigarettes in 2007, anti-smoking campaigns such as “*Tak Nak*” (“Say No”) from 2004–2011, and provision of smoking cessation services at government health clinics [15] have also been instituted. These measures, as well as changes in the country’s socioeconomic landscape are likely to have changed the smoking pattern of Malaysians over the past decade.

In the second National Health and Morbidity Survey (NHMS II) in 1996, a representative sample of the Malaysian population was surveyed, and of the 32,991 participants 24.8% reported being active smokers. Among males aged 18 years and older, almost half were current smokers (49.2%). Smoking was more common among respondents with low socioeconomic status and among those with primary school education. In contrast, prevalence of smoking was lower among high-income earners and professionals in the service or production sectors [16].

In this paper, we analyzed data from NHMS III in 2006 to determine the prevalence and socio-demographic factors associated with tobacco use among Malaysian men ten years after the previous survey. In addition, in this paper we discuss the effectiveness of current tobacco control measures in light of these findings.

## Methods

### Sampling procedure

We analysed data from the NHMS III. NHMS III was a nationwide, cross-sectional, population-based survey. The study sample was selected using a two-stage, proportional population size stratified sampling design and the Labour Force Survey (LFS) 2004 sampling frame from the Department of Statistics, Malaysia. The states constituted the primary strata with further urban–rural stratification. Enumeration Blocks (EBs) are artificially created and geographically contiguous areas consisting of 80–120 households and constitute the primary sampling units for the study. Living quarters (LQs) represent secondary sampling units. A total of 2,150 EBs (1425 urban and 726 rural) and 17,251 LQs were randomly selected. The number of households selected was based on 4.4 expected respondents per LQ. The total sample size was based on a previous finding of a 10% prevalence rate, margin of error of 1.2 and design effect of 2. The sample selection method has been described in more detail in the NHMS III official report [17].

Data was collected using a structured questionnaire administered by trained public health personnel. The questionnaire was developed by a committee of experts and pre-tested on a sample of respondents living in urban and rural areas of Klang, Bangsar and Sepang. These respondents were thereafter excluded from the sampling frame. The questionnaire was bilingual (Bahasa Malaysia and English) with additional translations of selected items and terminologies in Hokkien, Cantonese and Tamil, the dialects of the other two major ethnic groups in Malaysia. Data collection proceeded for four months from April to the end of July, 2006. Before each interview started, the interviewer read out the consent form in order to obtain written consent. To ensure a high response rate, unoccupied households were revisited up to three times. The study was approved by the Medical Research Ethics Committee, Ministry of Health, Malaysia.

### Measurements

Key socio-demographic status variables included age, gender, marital status, highest education level attained, occupation, residential locality and household income level. Smoking status was assessed by the following questions: “Have you ever smoked a cigarette in your lifetime?” followed by “In the last 30 days, how often did you smoke?”, “On average, how many cigarettes do you smoke per day?” Never smokers were individuals who never smoked in their lifetime; current smokers were those who smoked at least once in the last 30 days; ever smoker were those who had ever smoked but less than 100 sticks in their lifetime and ex-smokers were respondents who did not smoke in the past month but reported smoking 100 or more cigarettes in their lifetime. In our analysis, ex-smokers, ever smokers and never smokers were combined and constituted the non-smokers category.

Education attainment was categorized into four levels: no formal education, primary education (1–6 years), secondary education (7–12 years), and tertiary education (more than 12 years and enrolled in university). For types of occupation, respondents were categorised into 10 major groups according to the Department of Statistics standard method of classification, namely, senior officer, professional, technical, clerical, service, skilled agriculture and fisheries, craftsman, plant and machine operator, elementary occupation, and unemployed. Data on monthly household income was obtained using an open-ended question asking for the exact income which was later categorized into three categories: (a) less than RM 2000, (b) RM 2000–2999, and (c) RM 3000 and above.

### Data management and analysis

A total of 34,305 respondents aged 18 years and older were interviewed using a structured questionnaire. The overall response rate was 96.7%. We then analysed the smoking data on 15,639 male respondents. Double manual data entry method was employed for quality control. Analyses were performed using STATA version 10 and SPSS version 16. Data were weighted in the analysis to account for the complex study design and response rate. Descriptive statistics were used to estimate smoking prevalence and multivariable logistic regression to determine the influence of each variable on smoking status while simultaneously controlling for potential confounding effects by other variables. We report 95% confidence intervals without P values as the large sample size could generate significant results even if statistical differences or associations were small.

## Results

A total of 7,113 out of 15,639 respondents interviewed were current smokers (46.4%, 95%, CI 45.5–47.4). A majority of the smokers were between 21–40 years of age. The prevalence of smoking declined with age; with 59.3% (95% CI 57.4–61.2) among 21–30 year olds, 56.8% (95% CI 55.0–58.6) among 31–40 year olds, 48.5% (95% CI 46.7–50.3) among 41–50 year olds, and 35.0% (95% CI 32.9–37.1) among those aged 61 and above. There were fewer smokers among those with higher education attainment (31.4%, 95% CI 29.0–33.8), monthly household income of at least RM3000 (39.2%, 95% CI 37.2–41.2), and among working professionals (32.3%, 95% CI 29.6–35.1). Smoking was higher among the Malays (55.9%, 95% CI 54.8–57.1) and people of other indigenous ethnic groups (53.8%, 95% CI 51.2–56.3) than the Chinese (36.0%, 95% CI 34.1–37.9) and Indians (35.0%. 95% CI 32.0–38.0) (Table 1).

**Table 1:** Prevalence of smoking among Malaysian males aged 18 and above by socio-demographic variables

**Characteristics**	**Sample**	**Estimated population**	**Percentage of respondents who smoked (%)**	**95% CI**
Ethnicity				
Malay	8466	3114644	55.9	54.8–57.1
Chinese	3204	1278693	36.0	34.1–37.9
Indian	1193	474117	35.0	32.0–38.0
Other indigenous	1732	576818	53.8	51.2–56.3
Other	771	275161	55.0	50.6–60.3
Age group (Years)				
18–20	1192	437649	49.6	46.3–52.9
21–30	3285	1222328	59.3	57.4–61.2
31–40	3140	1166894	56.8	55.0–58.6
41–50	3180	1183816	48.5	46.7–50.3
51–60	2466	925384	40.8	38.8–42.8
61 and above	2103	773935	35.0	32.9–37.1
Marital status				
Single	3807	1414961	54.9	53.0–56.8
Married	11094	4129478	47.8	46.8–48.8
Divorce	199	73366	55.2	48.1–62.1
Widow/widower	191	71425	36.0	29.6–43.0
Education				
None	956	333280	48.5	45.2–51.8
Primary	5103	1864393	49.5	48.0–51.0
Secondary	7531	2824816	53.8	52.5–55.1
Tertiary	1640	645212	31.4	29.0–33.8
Monthly household income				
Less than RM 2000	9292	3351605	53.0	49.0–57.0
RM 2000–2999	2365	907525	47.7	45.6–49.9
> = RM 3000	3169	1251092	39.2	37.2–41.2
Occupation				
Senior officer	438	173326	34.5	30.1–39.0
Professional	1185	464081	32.3	29.6–35.1
Technical	1794	686112	44.6	42.3–47.0
Clerical	629	237063	47.8	43.9–51.8
Service	2950	1119877	52.2	50.3–54.2
Skilled agriculture and fisheries	2032	690104	58.2	55.8–60.5
Craft	1050	397210	58.4	55.0–61.6
Plant and machine operator	1775	666594	56.4	53.9–58.9
Elementary occupation	1199	424680	64.8	62.0–67.5
Unemployed	1510	549940	42.0	39.7–44.8
Residential area				
Urban	8843	3609890	45.2	44.0–46.4
Rural	6523	2106363	56.9	55.5–58.3

Table 2 shows the distribution of the age of smoking onset and number of cigarettes smoked per day among current smokers. A majority began to smoke before the age of 25 (90.1%), 19.7% (95% CI 18.8–20.6) between 13–15 year-old and 33.8% (95% CI 32.7–34.9) between 16–18 year-old. Only 5.6% of smokers smoked more than 20 cigarettes per day with 55.7% of male smokers smoking less than 11 cigarettes per day (Table 2).

**Table 2:** Distribution of male smokers by age of smoking initiation and number of cigarettes smoked per day

	**n (%)**	**Estimated population**	**95% CI**
Age started smoking (years)			
4–6	12(0.1)	4175	0.1–0.3
7–9	51(0.6)	18002	0.5–0.8
10–12	448(5.8)	164112	5.3–6.4
13–15	1501(19.7)	552322	18.8–20.6
16–18	2558(33.8)	949289	32.7–34.9
19–21	1869(24.3)	683068	23.3–25.3
22–24	442(5.8)	161312	5.3–6.3
25 and above	762(9.9)	276913	9.2–10.6
No. of cigarettes smoked per day			
1–5	1835(24.2)	661943	23.2–25.3
6–10	2338(31.3)	855871	30.3–32.4
11–15	1423(19.4)	530519	18.5–20.4
16–20	1420(19.4)	529399	18.4–20.4
21 and more	415(5.6)	154046	5.1–6.2

Cigarette were the main tobacco product used by Malaysian adult male smokers (92.7%, 95% CI 91.8–93.5) followed by clove cigarettes (44.6%, 95% CI 43.0–46.1) and hand-rolled cigarettes (38.1, 95% CI 36.4–39.9), less than 10% of smokers used bidis, pipe and shisha (Table 3).

**Table 3:** Type of tobacco product used by current adult male smokers in Malaysia

**Type of tobacco product**	**n (%)**	**Estimated population**	**95% CI**
Cigarettes	6491(92.7)	2399573	91.8–93.5
Clove	3074(44.6)	1109165	43.0–46.1
Hand-rolled tobacco	2670(38.1)	947303	36.4–39.9
Cigar	956(10.0)	245343	9.1–10.9
Pipe	418(6.3)	154264	5.6–6.9
Sisha	345(5.4)	132375	4.7–6.1
Bidis	232(3.4)	84746	3.0–4.0
Other type of tobacco product	15(0.7)	5542	0.4–1.2

Using multivariable logistic regression (Table 4), associations were observed between smoking status and level of education, occupation, ethnic group and age. Respondents without formal education were more likely to smoke than those with tertiary education (adjusted odds ratio (aOR) 2.09; 95% CI 1.67–2.60); and those who worked in skilled agriculture and fisheries sector were 2.00 (95% CI 1.66– 2.42) times more likely to smoke than senior officers. Respondents aged 21–30 year old were more likely to smoke compared to those aged 61 years and above (3.31; 95% CI 2.82–3.89). Respondents with monthly household income less than RM 2000 (1.27, 95% CI 1.13–1.43) were more likely to smoke than those with income of RM 3000 and above. Divorced (1.67, 95% CI 1.22–2.28) and residing in rural areas (1.12, 95% CI 1.03–1.22) were also associated with a higher likelihood of smoking.

**Table 4:** Association between socio-demographic factors and smoking among Malaysian males aged 18 years and above

	**Adjusted odds ratio**	**95% CI**
Residential Area		
Urban	1	
Rural	1.12	1.03–1.22
Education level		
None	2.09	1.67–2.60
Primary	1.95	1.65–2.30
Secondary	1.88	1.63–2.11
Tertiary	1	
Ethnicity		
Malay	2.29	1.98–2.66
Chinese	1.23	1.05–1.45
Indian	1	
Other indigenous	1.75	1.46–2.10
Other	1.48	1.15–1.91
Monthly household income		
Less than RM 2000	1.27	1.13–1.43
RM 2000-RM 2999	1.14	0.99–1.30
RM > =3000	1	
Marital status		
Married	1	
Single	1.05	0.93–1.18
Divorce	1.67	1.22–2.28
Widow/widower	0.87	0.63–1.20
Age group (years)		
18–20	2.36	1.90–2.94
21–30	3.31	2.82–3.89
31–40	2.85	2.47–3.28
41–50	1.93	1.69–2.22
51–60	1.32	1.15–1.51
61 and above	1	
Occupation		
Senior officer	1	
Professional	1.28	1.01–1.63
Technical	1.22	1.03–1.45
Clerical	1.22	0.98–1.51
Service	1.74	1.47–2.06
Skilled agriculture and fisheries	2.00	1.66–2.42
Craftsman	1.87	1.51–2.30
Plant and machine operator	1.85	1.53–2.22
Elementary	2.38	1.95–2.92
Unemployed	1.40	1.15–1.71

## Discussion

The prevalence of current smoking among adult males in this study (46.4%) is comparable to the findings by Rampal et al. (2006) [18] and the Malaysia NCD Surveillance-1 in 2006 [19], which found smoking rates of 47.2% and 46.5%, respectively. However, it is higher than rates from the Global Adult Tobacco Survey (GATS) in 2011 which reported that 43.6% of Malaysian male aged 15 years and above were current smokers [20].

Our study showed that the prevalence of smoking in Malaysia has dropped by only 2.8% between 1996 and 2006. The observed decrease, though statistically significant, is very modest compared to other countries that have reduced smoking prevalence by 9% to 25% over 10–20 years after implementing anti-tobacco measures [21,22]. This could be due to the fact that countries with more successful reduction in smoking prevalence quickly adopted the World Health Organization’s Framework Convention on Tobacco Control, while Malaysia has a shorter history of implementing tobacco control measures.

Other Asian countries which reported comparable smoking prevalence in adult males are Thailand (45.6%) [23] and Vietnam (50%) [24]. However, the prevalence of smoking was higher than India (24.3%) [25], but lower than China (66.9%) [26] and the Philippines (53.8%) [27]. These variations may be due to differences in the tobacco control programs and legislation implemented in these countries [28].

In the present study, the mean age of smoking onset (18.3 year old) was lower than reported by the NHMS II in 1996 (19.5 year old) (p <0.01) [16]. This is consistent with the mean age of smoking onset in Kuwait (18 years) [29] and Thailand (18.3 years) [23] yet is higher than India (17.8 years) [26]. In China, a three-year decrease in the mean age of smoking onset has been reported [30] and taken together with Malaysia, this may represent the success of the multinational tobacco companies which continue to focus their advertisement on the youngsters. Other plausible explanations would be the lack of perception of harmful effects of tobacco amongst Malaysians [31], higher affordability and easy availability and accessibility to tobacco products in Malaysia. Further investigations are certainly needed to elucidate the reasons for the decreased mean age of smoking onset in Malaysia while developed countries are show the opposite trend [32].

The present study found that 60% of Malaysian male smokers started smoking by the age of 18, which is higher than those reported in China (52.7%) [26], but lower than in the USA (80%) [33] and Canada (82.6%) [34]. This heralds a worrying trend in Malaysia since at present more than a quarter of its population is aged 15 and below [35]. Health education (particularly on smoking) should be incorporated as a subject in primary and secondary schools curricula and should include encouraging healthy leisure time activities to deter smoking, raising awareness of health impact of smoking, enhancing children’s emotional intelligence and social skills to resist smoking. Schools should have trained personnel who are able to provide smoking cessation counseling to students. Emphasis should be on rehabilitation and prevention of smoking instead of punishment as commonly practiced now. More severe penalties such as higher fines, revocation of business license or jail term should be meted out to those who sell cigarettes to minors.

The average number of cigarettes smoked by the study respondents was 12.3 cigarettes per day, slightly less than 13.3 cigarettes per day in 1996 (p < 0.01) [16]. Compared to neighboring countries, this average number of cigarettes is more than the 11.3 cigarettes per day reported by the Philippines [27] yet lower than 13.5 and 14.3 cigarettes per day reported in Vietnam [24] and China [36], respectively. The decrease in the average number of cigarettes smoked may reflect an actual decrease in the demand amongst Malaysian men or an increase in the price of tobacco products in the country over the past five years. For example, with respect to increasing tobacco prices, a Taiwanese study reported the possibility of decreasing the country’s average annual per capita cigarette consumption by 14.86 packs with a 44% increment in the price of cigarettes [37]. The present study also found that 56.0% of current male smokers smoked less than 11 cigarettes per day and another 38.8% smoked between 11–20 cigarettes per day. Intensified efforts directed at encouraging smokers to quit smoking are therefore encouraged by these trends and findings.

The most commonly consumed tobacco product among Malaysian male smokers was the cigarette (90%) and hand-rolled tobacco (40%). Cigarettes were also the most consumed tobacco product in the Philippines (97.8%) [27] However, cigarettes were less popular in Thailand (64.9%) and India (43.1%) where hand-rolled tobaccos were also commonly used [23,26]. Currently, the tobacco control policies are more focus on cigarettes than other tobacco product. However the sales of hand-rolled cigarettes also need to be regulated considering that a significantly high proportion of smokers also use them.

Male respondents in rural areas were more likely to be smokers compared to males living in urban areas (Adjusted Odds Ratio (aOR) 1.12, 95%, CI 1.03–1.22). This finding is consistent with findings from the NHMS II [16] and with findings from a population-based study in China, Thailand and Korea [36,38,39], yet runs contrary to reports from the western countries [40,41]. Several possible explanations for this finding are, urban residents are more often exposed to anti-smoking campaigns and measures, and the co-existence of other known risk factors for smoking, namely lower income and education levels, among those living in rural areas. However, the observed association between urban–rural and smoking status was maintained after adjusting for income and education, thereby excluding them as the possible explanation for the difference. Another possible contributing factor for higher prevalence of smoking in rural areas is targeted marketing by the tobacco industry towards the rural residents [42]. Furthermore, tobacco-growing contributes substantially to the economy of many rural communities in Malaysia and thereby makes smoking a more accepted behaviour in this setting.

A lower smoking prevalence was found among men aged 50 years and above in this study, which is in accordance with estimates from Korea [43] (50.0% among 50–55 years old compare to 62.0% among age group of 30–34 years old) and Albania [44] (26.5% among respondents age 55–64, 40.9% among 45–54 years old compared to 58.7% among those age 25–34 years old). The higher proportion of ex-smokers in older age groups was among the plausible explanations to the present finding. The proportion of ex-smokers according to age group were as follows: 17.1% (95% CI 14.5–20.1) among respondents aged 18–20 years old, 14.5% (95% CI 13.1–16.1) among 21–30 years old, 18.9% (95% CI 17.2–20.6) among 31–40 years old, and 27.2% (95% CI 25.2–29.2), 36.1% (95% CI 33.7–38.5) and 45.8% (95% CI 43.1–48.1) among 41–50, 51–60 and 61 years and above respectively. A similar pattern was reported by the Global Adult Tobacco Survey (GATS), which found that in Malaysia the proportion of current smokers was higher among the 25–44 years old age group (54.9%), decreased to 43.8% for 45–64 year olds, and decreased further to 25.3% for respondents 65 years and above [20]. Possible explanations for this include having more time to encounter smoking-related health problems, increased health consciousness with age, more time to be exposed to anti-smoking efforts, and a sense of vulnerability that is less pronounced in the younger age groups. This sense of vulnerability, in addition to having a greater likelihood of experiencing adverse health events from smoking, tend to make older males more receptive to public health messages and medical advice, and therefore more likely to quit smoking [45]. The finding that older Malaysian males smoke less than middle-aged and younger males is also a cause for concern for the two younger cohorts, as population trends show that there will likely be a rise in their numbers in the future, and therefore a greater burden on the country from smoking unless these trends are reversed.

Divorced men were found to smoke more than single, married or widowed men. This is consistent with findings reported by Nystedt in 2006 [46], Cho *et al*. in 2008 [47], and Aekplakorn *et al*. in 2008 [38]. There were no significant differences in smoking prevalence between married and single males, and this is corroborated by Yu *et al*. [48] who reported smoking was not related to marital status among adult persons residing in urban areas of China. These findings may be explained by the ‘marriage protection’ and ‘marriage selection’ theories [49], which posit that emotional distress due to divorce cause divorcees to turn to smoking for relief. Moreover, married people tend to have more economic advantages, and receive more social and psychological support which can make quitting smoking more likely. It is also possible that the healthier non smokers are more likely to get and stay married than those who are divorced as posited by the marriage selection theory.

More than half of the Malays and other indigenous ethnic groups were current smokers. Malays and other indigenous ethnic groups were more likely to be active smokers compared to Indians. Similar proportions have been observed in the previous NHMS surveys [16]. Potential factors contributing to the association between ethnicity and smoking status are many and deserve further investigation, especially as adjustment for age, socio-economic status and other factors did not remove the effect of ethnicity on smoking status.

A significant association was observed between both monthly household income and education level with current smoking status. The higher the level of an individual’s education and income, the less likely that individual is to be a current smoker. Interestingly, a study from Saudi Arabia showed that smoking prevalence was significantly higher among literate than illiterate Saudis [50]. The authors postulated that anti-smoking campaigns unintentionally conveyed smoking as a behaviour of those with elevated social status and therefore smoking was more popular among those who were exposed to those advertising campaigns. In this study, however, the findings are more consistent with what one would predict, that the better educated one is, the more concerned one would be about health and therefore the lesser the likelihood to smoke. Other studies by Huisman *et al*. in 2005 and Gilmore *et al*. in 2000 [51,52] reported higher income level as a protective factor for smoking while Aekplakorn *et al*. (2008) [38], Cho *et al*. (2008) [47], Lim *et al*. in 2010 [39]. Khang and Cho (2006), [53] found that the likelihood of smoking was higher among those from lower education and income bracket.

Type of occupation was significantly associated with current smoking status in the present study. Elementary workers and agricultural workers had a higher tendency to smoke than those in management and other professional occupations. This finding is consistent with what has been reported from previous studies in Europe and Asia [39,54-56] where it has been postulated that lower-level occupational groups face more physical and psychosocial stressors compared to the managerial and professional classes and therefore, are more likely to engage in high risk health behaviors such as smoking.

In accordance with results linking occupation and current smoking status, results from this study showed that Malaysians with low socioeconomic status had greater smoking rates than those with high socioeconomic status. This pattern is similar to other countries. It is likely that individuals with low education, low level occupation, and low income have less access to adequate health care information and face financial difficulties that increase their stress levels, making them more susceptible to partake in unhealthy lifestyle or health risks such as smoking. Previous studies have shown that smoking is often used as a coping mechanism to deal with stress [57]. Therefore, social disparities in smoking need to be addressed into future health policies.

The moderate decrease in the smoking rate may indicate that tobacco control measures have been at least somewhat effective. Since the introduction of the Control of Tobacco Products Regulation in 1993, the prevalence of exposure to secondhand smoke in gazetted areas has reportedly declined while prohibition of smoking in Malaysian homes has increased from 7% in 2005 to 40.3% in 2009, with nearly half of all smokers designating their homes as non-smoking areas. The percentage of smokers working indoors who refrained from smoking at work increased from 33.6% in 2005 to 64.4% in 2009 [11].

Pictorial health warnings were introduced on cigarette packaging in 2009. Reading the warning labels closely often or very often increased from 54.4 to 68.3% and giving up a cigarette at least once due to the reading the label increased from 24.1% to 45.4% [11]. Beyond these self-reported statistics however, the broader impacts of the pictorial warning on smoking prevalence is not known and requires further investigation.

The effect of banning tobacco advertising from the mass media in 2004 has also been unclear. The International Tobacco Control Survey in 2009 [11] reported that only 9% adult smokers noticed clothing or other items linked to a cigarette brand. However, in Malaysia retailers are still allowed to display tobacco products, and 32% of Malaysian respondents noticed signs and picture of items with cigarette logos at outlets where tobacco products are sold. Regulations prohibiting the sale of tobacco products to minors are in place, but lack of enforcement has resulted in an increase in cigarette purchases by under-aged adolescents in spite of the law [58]. In 2004, the Ministry of Health launched a five-year national anti-smoking campaign with the slogan ‘Tak Nak’ (Say No) that reached more than 92% of the population. The campaign apparently succeeded in educating the public on the dangers of smoking [11].

Cigarette prices in the country have risen as an effect of high tobacco taxes [11]. Imposing higher taxes on tobacco products may be an effective strategy to boost smoking cessation. Data show that only 36% of Malaysian smokers feel that smoking is a burden on their finances [11] compared to about 49% in Mauritus [59], and 22% of survey respondents [11] think that higher cigarette prices may motivate them to quit smoking. Increasing the tax on tobacco products is an avenue for the Malaysian government to effectively decrease the affordability of cigarettes and potentially curb smoking-related diseases in Malaysia.

This study has several limitations; principal among them is its cross-sectional design which limits study findings to the reporting of associations between current smoking status and exposure. In addition, smoking status is based on self-reporting and is not validated by objective measurements like biochemical markers of smoking. This could result in underestimating the actual prevalence of smoking amongst Malaysian males, but self-reported data on cigarette smoking and smokeless tobacco use has been found to be valid in other surveys [60]. The strengths of this study include its large sample size and representativeness of the Malaysian population. Moreover, the interview technique employed was standardized and made use of a personalized approach to ensure a high response rate (96.7%) and greater willingness to report on socio-economic status and education level, both key factors for examining the relationship between social class and smoking in the adult male population in Malaysia.

## Conclusion

In conclusion, this study reports and comments on the ongoing high prevalence of current male smokers in Malaysia. It raises awareness that unless public health efforts are taken broadly and comprehensively, tobacco will remain a primary contributor to premature morbidity and mortality in Malaysia. Public health policies and action need to focus on high risk sub-populations identified through this survey, principally younger, rural, Malay males from a lower income group, and with less formal education.

## Competing interests

The authors declared that they have no competing interest.

## Authors’ contributions

LKH wrote the manuscript, and carried out statistical analysis with assistance from SMG and KCC, AFY, ZZ, MHNM and SS responsible for data collection, design and coordination of the study. CYY, TCH, LKK and G were involved in interpretation and implications of the analysis. All authors contributed to developing the manuscript, and read and approved the final version.

## Pre-publication history

The pre-publication history for this paper can be accessed here:

http://www.biomedcentral.com/1471-2458/13/8/prepub
